# The spectrum of radiological findings in neurocryptococcosis: case series and systematic review

**DOI:** 10.1590/0100-3984.2024.0107

**Published:** 2025-05-01

**Authors:** Anna Luisa Silva Campos, Kezia de Souza Pinheiro, Matheus Leite Rassele, Marcos Rosa-Júnior

**Affiliations:** 1 Department of Neuroradiology, Hospital Universitário Cassiano Antônio de Moraes da Universidade Federal do Espírito Santo/Empresa Brasileira de Serviços Hospitalares (HUCAM-UFES/EBSERH), Vitória ES, Brazil; 2 Hospital Meridional Vitória/Kora Saúde, Vitória, ES, Brazil; 3 Santi Medicina Diagnóstica, Vitória, ES, Brazil

**Keywords:** Cryptococcosis, Central nervous system, Magnetic resonance imaging, Criptococose, Sistema nervoso central, Ressonância magnética

## Abstract

This study involved a retrospective analysis of nine cases of neurocryptococcosis (eight from our institution and one from another institution) seen between May 2014 and May 2022, together with a systematic review of the literature indexed in the PubMed, Embase, and Lilacs databases. Clinical and radiological features of those cases were further refined via an additional comprehensive literature review. The following search string was employed: cryptococcosis AND central nervous system AND (magnetic resonance imaging OR X-ray computed tomography). The search was limited to articles published between July 1978 and May 2022. Two authors, working independently, searched for and selected studies that met the inclusion criteria, and another author reviewed conflicts in a blinded manner. We used Rayyan.ai software to organize the studies, and the review was structured in accordance with the 2020 Preferred Reporting Items for Systematic reviews and Meta-Analyses guidelines. Understanding the prevalence of different patterns of neurocryptococcosis is crucial for improving diagnosis and supporting decision-making in clinical practice. Our review of the literature demonstrated that imaging examinations are a valuable resource for early diagnosis, as well as for assessment of the initial extent and pattern of the disease.

## Abstract (pt)

Este estudo envolveu uma análise retrospectiva de nove casos de neurocriptococose (oito de nossa instituição e um caso de outra instituição) observados entre maio/2014 e maio/2022 e uma revisão sistemática da literatura realizada nas bases de dados PubMed, Embase e Lilacs. As características clínicas e radiológicas desses casos foram refinadas por meio de uma revisão abrangente adicional da literatura. Foram analisados os artigos relacionados à seguinte descrição: “cryptococcosis AND central nervous system AND (magnetic resonance imaging OR X-ray computed tomography)” publicados entre julho de 1978 e maio de 2022. Dois autores pesquisaram e selecionaram, de forma independente, os estudos que atendiam aos critérios de inclusão, e outro autor revisou os conflitos às cegas. Usamos o *software* “Rayyan.ai” para organizar os estudos, e a revisão foi estruturada seguindo as diretrizes de 2020 do Preferred Reporting Items for Systematic reviews and Meta-Analyses. Entender a prevalência de diferentes padrões de neurocriptococose é crucial para melhorar o diagnóstico e apoiar a tomada de decisão na prática clínica. Após revisão da literatura, entende-se que os exames de imagem são um recurso valioso para o diagnóstico precoce, avaliação da extensão inicial e padrão da doença.

## INTRODUCTION

Cryptococcosis is an infectious disease caused by fungi of the species *Cryptococcus neoformans* and *Cryptococcus gattii*; it is generally pathogenic in individuals with compromised immune systems, such as patients with AIDS, especially those with a CD4 cell count < 100 cells/mm^**(^1^)**^. Cryptococcus represents the main pathogen of fungal meningitis and the third most common intracranial pathogen, surpassed only by HIV and *Toxoplasma gondii*^**(^2^)**^. The most common route of infection is through inhalation of fungal spores, which can lead to hematogenous dissemination to the central nervous system (CNS), resulting in generalized subacute meningitis^**(^1^)**^.

The most common clinical manifestations of CNS cryptococcal infection include headache, nausea, fever, meningismus, mental confusion, seizures, visual symptoms, and even focal neurological deficit. The diagnosis of CNS fungal infection should be considered in individuals with any of these manifestations, especially in those who are immunocompromised^**(^3^)**^.

The major environmental sources of *C. neoformans* include soil contaminated with pigeon excreta (*C. neoformans* var. *neoformans* and *C. neoformans* var. *grubii*) and eucalyptus trees/decaying wood (*C. neoformans* var. *gattii*). Whereas *C. neoformans* var. *gattii* is found mainly in tropical and subtropical regions, *C. neoformans* var. *neoformans* is found worldwide. *C. neoformans* var. *neoformans* usually infects immunocompromised individuals, leading to acute diffuse meningitis or meningoencephalitis. In contrast, infection with *C. neoformans* var. *gattii* more typically manifests as a granulomatous inflammatory response in immunocompetent hosts^**(^4^)**^.

Regarding imaging examinations, neurocryptococcosis produces a wide variety of magnetic resonance imaging (MRI) findings that may vary depending on the immunological status of the patient, some of which have been described in the literature, such as hydrocephalus, leptomeningeal enhancement, enlarged perivascular spaces, plexitis, and cryptococcoma, which may occur isolation or concomitantly^**(^3^)**^. In this context, it is essential to understand the importance of imaging examinations in the diagnosis, differential diagnosis, and monitoring of the treatment response in cases of neurocryptococcosis, in immunocompromised and immunocompetent patients alike.

The aim of this study was to carry out a systematic review of the literature to identify the patterns of involvement that can support the diagnosis of neurocryptococcosis on imaging examinations and to exemplify those patterns through examinations performed at our institution.

## METHOD

This was a retrospective observational study with a critical analysis of the literature, synthesizing the results of several primary and non-primary studies, using images of patients from a hospital in the Brazilian state of Espírito Santo. All of the patients whose images are presented in the study had a confirmed diagnosis of cryptococcosis. The study was approved by the local research ethics committee (Reference no. 48412221.3.0000.5071).

We performed a retrospective analysis of nine different cases of neurocryptococcosis (eight from our institution and one from another institution), all seen between May 2014 and May 2022, together with a systematic literature review following the 2020 Preferred Reporting Items for Systematic reviews and Meta-Analyses (PRISMA) guidelines, using the Embase, Lilacs, and PubMed databases to identify articles published between July 1978 and May 2022. Clinical and radiological features of the cases were further refined via an additional comprehensive literature review.

The original text was translated from Portuguese to English with the assistance of the artificial intelligence tool ChatGPT-4, developed by OpenAI, and was reviewed by human authors.

### Data sources

All data were collected retrospectively from three databases: PubMed, Embase, and Lilacs. The search strategy was based on US National Library of Medicine Medical Subject Headings, and we employed the following search string: Cryptococcosis AND Central Nervous System AND (Magnetic Resonance Imaging OR X-Ray computed tomography).

### Study selection

Two authors, working independently, searched for and selected studies that met the inclusion criteria, and another author reviewed conflicts in a blinded manner. We used Rayyan.ai software to organize the studies, and the review was structured in accordance with the PRISMA guidelines.

The articles identified were initially included or excluded after analyzing the titles and abstracts. The articles remaining at the end of these preliminary analyses were fully evaluated. Duplicates were excluded, as were studies that did not focus on cryptococcosis, studies without imaging findings, those evaluating irrelevant populations (animals) and with incorrect populations (*in vitro* or animals).

**Figure 1:**
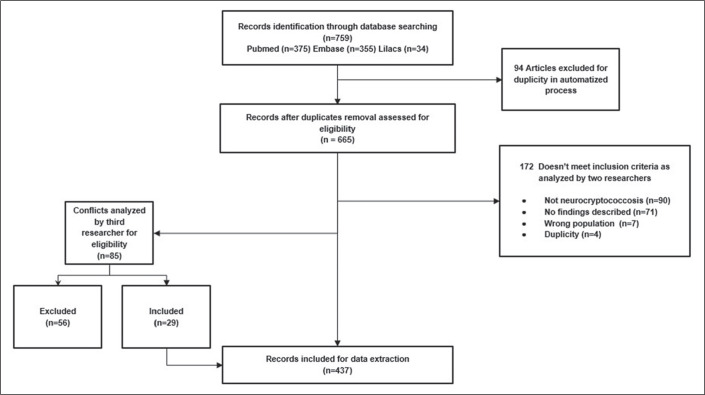
Flow chart of the article selection process.

As can be seen in the PRISMA flow chart (Figure 1), the initial search returned 759 articles related to the topic according to the search terms: 375 from PubMed; 355 from Embase; and 34 from Lilacs. Of those, 94 were excluded by the software because they were duplicates and 172 were excluded by the criteria mentioned above (four of those because they were duplicates missed in the first pass). Therefore, we initially evaluated a total of 493 articles. Among those, there were 85 articles for which there were conflicts (differences of opinion regarding their suitability for inclusion), and those articles were analyzed by another author who was blinded to which author had which opinion, with 56 of the 85 articles being excluded. That reduced the total to 437 articles. However, there were 21 articles for which the full texts were not available. Therefore, our analysis included 416 articles that were evaluated in their entirety.

The statistical analyses were performed in Microsoft Excel. The studied factors were analyzed in terms of their frequency.

### Image search

Most of the findings of interest that were listed in the survey stage were actively researched using the word search tool of the radiology information system employed at our hospital (CLINUX; Genesis Tecnologia, Vitória, Brazil) to filter for the presence of these findings in the reports issued for the respective examinations. In addition, images of some findings were provided by collaborators of the work, from their own files.

All images meeting the criteria above were collected and organized by the researchers, after which each image was analyzed together with a neuroradiologist with 14 years of experience and images with the findings of interest were selected. After the relevant images had been selected, we anonymized each examination to remove personal and sensitive data, using the 2022 RadiAnt DICOM Viewer (https://www.radiantviewer.com; Medixant, Poznań, Poland).

## RESULTS

The articles analyzed were categorized according to the type of study as described in Table 1.

**Table 1:** Types of articles included in this review.

Article type	n
Case report	245
Case series	74
Review	61
Cohort study	14
Case-control study	9
Pictorial essay	9
Book chapter	1
Author comment	1
Editorial	1
Translational study	1

We classified the radiological findings on the basis of the image processing techniques used, image pattern, enhancement pattern, and location of the lesions. We also collected data related to epidemiological factors such as sex, immunological status, HIV status, and use of highly active antiretroviral therapy (HAART).

In our epidemiological analysis, impasses were encountered, because many articles described the number of radiological findings but not the size or even the characteristics of the study population. In this context, we categorized data from the articles that described those characteristics and found the following epidemiological data:

• Sex (data available for 516 patients): male, n = 323; female, n = 158; no data, n = 35.

• Immune status (data available for 555 patients): immunosuppressed, n = 179; immunocompetent, n = 234; no data, n = 142.

• HIV status (data available for 553 patients): positive, n = 87; negative, n = 348; no data, n = 118.

• Use of HAART (data available for 587 patients): yes, n = 56; no, n = 389; no data, n = 142.

Regarding the spectrum of radiological presentations (Table 2), a total of 36 patterns were found, chief among which were normal examination, meningitis, hydrocephalus, dilated perivascular spaces, cryptococcomas, cysts, and lacunar infarcts. Other less common patterns are also described in Table 2, as is the total number of each finding. Figure 1 shows the article selection process.

**Table 2:** Patterns seen on imaging of cases of neurocryptococcosis.

Pattern	n
Meningitis	657
Hydrocephalus	505
Dilation of perivascular spaces	429
Cryptococcoma	306
Lacunar infarction	207
Normal examination findings	183
Brain atrophy	168
White matter lesion	130
Brain edema	80
Ventriculitis	53
Encephalitis	52
Unspecified infarction	48
Mass/nodules	48
T2 hyperintensity	47
White matter lesion	37
Foci of hypodensity	30
Abscess	27
Ependymitis	17
Cryptococcal immune reconstitution inflammatory syndrome	15
Plexitis	15
Basal exudates	13
Cortical infarction	12
Extra-axial cyst	8
Arachnoiditis	9
Restricted diffusion	5
Granuloma	5
Pseudocyst	5
Cyst	4
Cranial nerve enhancement	4
Hemorrhage/hematoma	4
Vasculitis	3
Calcification	3
Sinusitis	3
Cyst with eccentric nodule	3
Myeloradiculitis	2
Grouped extra-axial cysts	1
Unclassifiable	47

## CASE SERIES

As previously described, images were collected to exemplify the most common patterns and some rare patterns of involvement, as described below.

### Leptomeningitis

**Figure 2:**
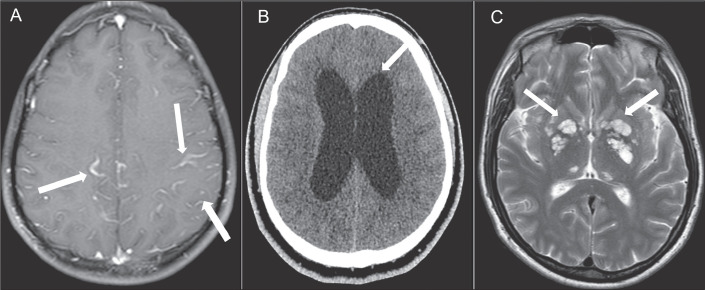
MRI scans of three different patients. A: Meningitis. Contrast-enhanced axial T1WI showing leptomeningeal enhancement. B: Hydrocephalus. Axial computed tomography scan demonstrating dilation of the lateral ventricles. C: Dilation of perivascular spaces. Axial T2WI showing enlarged perivascular spaces with a soap-bubble appearance.

*Cryptococcus* spp. typically gain access to the CNS parenchyma secondarily via the hematogenous route, penetrating the walls of the meningeal vessels through perivascular spaces connected to the subarachnoid spaces and then causing a meningeal infection in almost all patients^**(^5^)**^, which results in leptomeningeal enhancement on contrast-enhanced T1-weighted imaging (T1WI), as illustrated in Figure 2.

### Hydrocephalus

Hydrocephalus (Figure 2) can be communicating or noncommunicating. The factors that contribute to the tendency of individuals with cryptococcal meningitis to develop hydrocephalus are not fully understood. However, some patients experience a gradual onset of meningitis that progressively becomes hydrocephalus over weeks to months. In cases of chronic meningitis, obstruction of the cerebrospinal fluid circulation is often attributed to inflammation affecting the meninges at the base of the brain^**(^6^)**^.

### Dilation of perivascular spaces

After penetrating the CNS through the meningeal vessels, the fungus migrates to the Virchow-Robin (perivascular) spaces, which subsequently dilate after the activation of inflammatory cells and the deposition of mucoid material^**(^3^)**^, resulting in enlarged perivascular spaces on T2WI (Figure 2).

### Cryptococcoma

**Figure 3:**
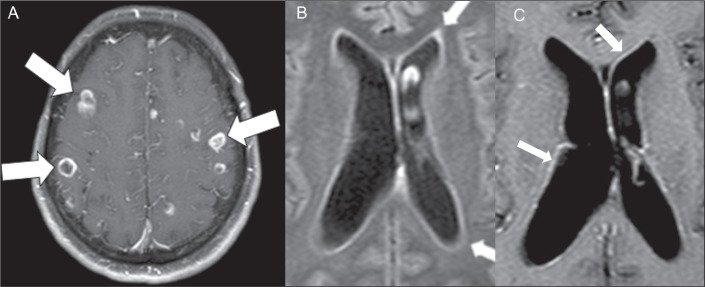
MRI scans of two different patients. A: Cryptococcomas. Contrast-enhanced axial T1WI showing various cerebral cryptococcomas with ring enhancement. B,C: Ventriculitis. Axial T2-weighted fluid-attenuated inversion recovery sequence (B) and contrast-enhanced T1WI (C) demonstrating the ventriculitis as a line of faint signal hyperintensity on a fluid-attenuated inversion recovery sequence and contrast enhancement of the walls of the lateral ventricles.

Cryptococcomas are mass lesions that can appear in the cerebrum (Figure 3), brainstem, or cerebellum, and may or may not present contrast enhancement. On MRI, they exhibit a variety of features, from hyperintensity on T2WI to a lack of enhancement on T1WI. Given the diagnostic complexity, it is not uncommon for cryptococcomas to be initially misinterpreted as neoplasms, abscesses, neurocysticercosis, or dilated perivascular spaces. However, cryptococcomas can develop in multiple locations within the brain parenchyma because of infiltration by the fungus^**(^7^)**^.

As a marker of fungal infection, trehalose is specific, although not highly sensitive. Using spectroscopy, Luthra et al.^**(^8^)**^ found a trehalose peak in cryptococcoma walls and cerebral mucormycosis in five of the eight patients evaluated, where it appeared as multiple signals ranging from 3.6 ppm to 3.8 ppm. That profile, especially the trehalose peak, suggests a diagnosis of fungal infection.

### Ventriculitis

Ventriculitis presents as a line of hyperintensity and enhancement around the ventricle on gadolinium contrast-enhanced fluid-attenuated inversion recovery MRI sequences (Figure 3). It is an uncommon CNS infection that has been referred to by a variety of terms, including ependymitis, intraventricular abscess, ventricular empyema, and pyocephalus. That variety reflects various facets of the pathological process. There are multiple routes by which a pathogen can infiltrate the intraventricular system, including direct implantation as a secondary result of trauma or neurosurgical procedures such as ventricular catheter placement; contiguous extension, such as the rupture of a brain abscess; and extension to the ventricles with hematogenous spread to the subependymal region or choroid plexus. On MRI, the ventricular walls often show high signal intensity on T2WI, whereas the ventricles themselves can be seen to be dilated, often containing debris. An additional finding can be choroidal plexitis^**(^9^)**^.

### Restricted diffusion in the cerebral cortex

Up to 4% of patients with cryptococcal meningitis may present with secondary cerebral infarction that typically affects the cortex but can involve the basal ganglia, thalamus, and internal capsule. The development of vasculitis, inflammation, or thrombosis in the cerebral blood vessels can lead to these infarcts.

**Figure 4:**
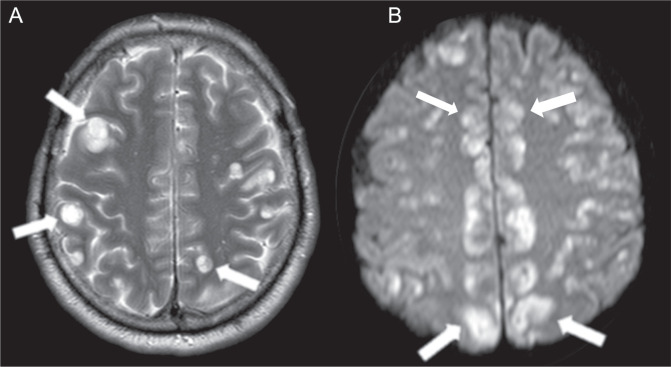
MRI scans of two different patients. A: Axial T2WI demonstrating intraparenchymal cysts. B: Diffusion-weighted imaging showing restricted diffusion in the cerebral cortex.

The findings for cerebral infarcts on MRI have been delineated as restrictive lesions on diffusion-weighted imaging and hypointensity on apparent diffusion coefficient maps. Cerebral infarctions are categorized as one of two distinct types: lacunar; and large (in the territories of the anterior, posterior, or middle cerebral arteries). In addition, foci of restriction can also result from the leptomeningeal inflammatory process that irritates the cerebral cortex due to its proximity, leading to encephalitis^**(^10^)**^ (Figure 4).

### Cryptococcal immune reconstitution inflammatory syndrome

**Figure 5:**
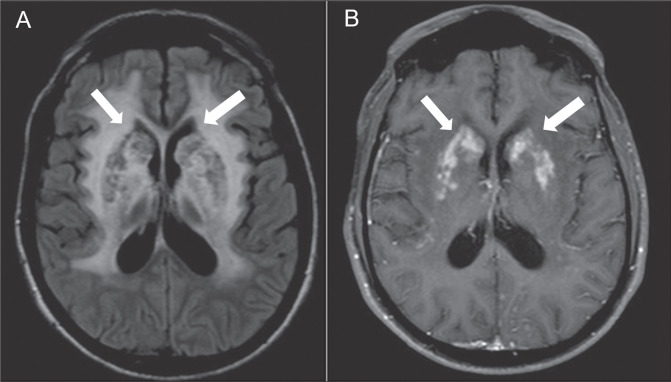
MRI scans of a patient with C-IRIS. Fluid-attenuated inversion recovery (A) and contrast-enhanced T1WI (B) demonstrating impregnation of the basal ganglia.

Cryptococcal immune reconstitution inflammatory syndrome (C-IRIS) is a rare and contradictory inflammatory reaction, occurring after treatment or subclinical infections, following exaggerated restoration of the immune response to specific antigens or pathogens. Treatment with HAART influences the interpretation of neuroimaging features related to opportunistic infections in patients with HIV/AIDS, especially regarding CNS cryptococcosis and recognition of the C-IRIS phenomena. One characteristic sign is the presence of focal meningeal and parenchymal gadolinium enhancement, which tends to be particularly conspicuous along the convexities of the cerebral hemispheres, often accompanied by underlying parenchymal inflammatory edema and enhancement. Other indicators of C-IRIS include linear perivascular enhancement in fissures, choroid plexus enhancement, and enhancement of dilated Virchow-Robin space pseudocysts^**(^11^)**^, as shown in Figure 5.

### Cysts and cystic lesions with the dot sign

**Cysts.** To our knowledge, there have been no studies describing the real pathophysiology of intraparenchymal cysts in cryptococcosis. However, it is believed that the fungus can migrate through the perivascular spaces and reach topographies more distant from the capsular region, which may appear on the image as intraparenchymal cysts best seen on T2WI (Figure 4A).

**Figure 6:**
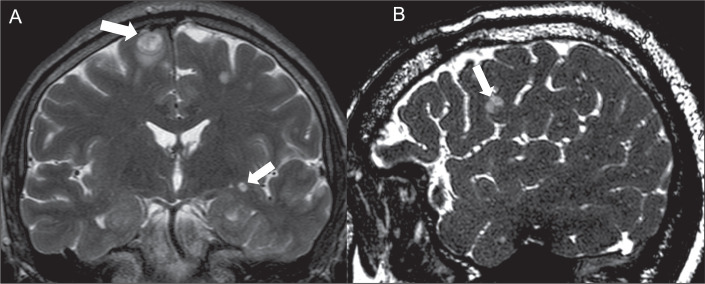
MRI scans of a patient showing cystic lesions with the dot sign. Coronal T2WI (A) and sagittal three-dimensional T2WI (B) demonstrating cystic lesions with the eccentric nodule, mimicking neurocysticercosis.

**Cystic lesion with the dot sign.** The dot sign is seen when there is a nodule within a cystic lesion, being a pattern typically considered pathognomonic of neurocysticercosis in its vesicular phase, although cryptococcosis can mimic that (Figure 6). However, in neurocysticercosis, the dot sign rarely shows gadolinium enhancement, thus distinguishing it from cryptococcal encephalitis^**(^12^)**^.

### Vertebral and spinal cord lesions

Spinal involvement is an uncommon manifestation of cryptococcosis and can be intradural or extradural. Intradural involvement comprises two subtypes: extramedullary lesions, mainly arachnoiditis, with or without mass formations; and intramedullary lesions, mainly cryptococcomas, granulomas, and abscesses. Lesions show an isointense or mildly hyperintense signal on T1WI and a hypointense signal on T2WI, together with edema in the surrounding area and solid or ring enhancement after contrast injection.

**Figure 7:**
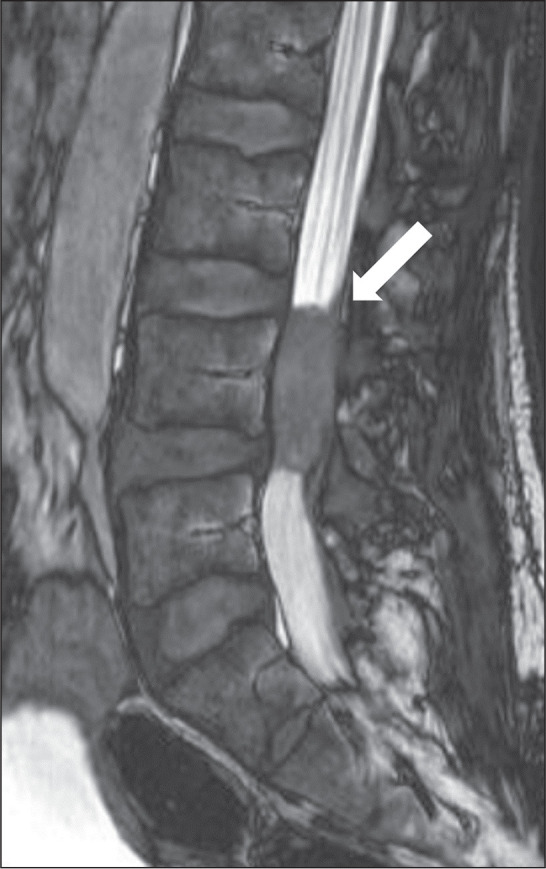
Lesion within the spinal canal. Sagittal T2WI demonstrating an expansile lesion within the spinal canal, mimicking an intradural neoplasm.

Typically, hyperintensity on T1WI arises because of fibrosis and inflammatory cellular infiltrates in the granulomatous tissue. Although hyperintensity on T1WI may indicate intramedullary cryptococcoma, it is not pathognomonic, given that similar findings have been reported in several neoplastic and granulomatous conditions. Lesions mainly appear in the thoracic or upper lumbar region (Figure 7) and have an average size smaller than that of a vertebral body. A conclusive diagnosis requires additional confirmation through microscopic identification of cryptococci or detection of cryptococcal antigen in cerebrospinal fluid^**(^13^)**^.

There have been a few reports of cases of cryptococcosis with spinal cord involvement with distinct patterns^**(^14^)**^: including the medullary cone (T2WI hyperintensity related to edema); involving the cervical spine (T2WI hyperintense lesions with cord expansion and prominent gray matter hyperintensity); longitudinal extensive transverse myelitis in the thoracic segment; and granuloma/cryptococcoma (localized, solid, tumor-like mass presenting T1WI hypointensity with a contrast-enhancing rim), which is a differential diagnosis of a spinal tumors.

## DISCUSSION

Cryptococcosis is a disease caused by encapsulated yeasts of the genus *Cryptococcus*, mainly by the species *C. neoformans* and *C. gattii*, and is found in animal excrement, especially that of birds and some mammals^**(^15^)**^. It is a disease that affects people living with HIV or other immunocompromised individuals, such as organ transplant recipients, patients on prolonged glucocorticoid therapy, and patients with hematological malignancies, although it can also be seen in immunocompetent patients, in whom an underlying predisposing factor may not be apparent^**(^16^)**^. Most of the patients evaluated in the articles included in this review had not tested positive for HIV infection, which could be explained by publication bias, which is the tendency to publish what is rarer, which, in this context, is the disease affecting immunocompetent patients.

In patients infected with HIV, susceptibility to various opportunistic infections is largely influenced by the level of T cell-mediated immune suppression. Since the introduction of HAART in 1987, there has been a significant reduction in the incidence of HIV-related opportunistic infections in the CNS. However, several factors influence the scenario of CNS infections associated with HIV, such as lack of knowledge about the disease itself, as well as resistance and lack of adherence to HAART^**(^17^)**^.

Among the fungal infections that most affect the CNS are aspergillosis, cryptococcosis, mucormycosis, and candidiasis^**(^15^)**^. In the case of neurocryptococcosis, the main initial site of involvement is the respiratory tract, from which the yeasts spread hematogenously to the CNS, penetrate the walls of the meningeal vessels, and migrate to the Virchow-Robin (perivascular) spaces, which consequently dilate following the activation of inflammatory cells and the deposition of mucoid material. Once the fungus crosses the blood-brain barrier, the CNS offers a favorable environment for fungal multiplication due to the existence of specific neuronal substrates used in order to generate melanin, protecting them against oxidative stress, phagocytosis, and antifungal agents^**(^3^)**^.

Neuromycoses have been increasingly diagnosed because of the rise in the incidence of HIV/AIDS, better critical care for patients with serious illnesses, advances in neuroimaging, and the application of microbiological techniques that are more sensitive^**(^18^)**^. The exact diagnosis is made through microbiological investigations, including culture of cerebrospinal fluid, identification of the fungus with India ink staining, and determination of latex agglutination antigen titers in cerebrospinal fluid and blood^**(^18^)**^.

Regarding imaging, each organism has typical characteristics that help refine the differential diagnosis and, in some cases, allow specific diagnoses^**(^15^)**^. Although the radiographic features of the disease are variable and often nonspecific, understanding the imaging appearance of CNS fungal infections is imperative because early diagnosis facilitates treatment of these infections, which are rapidly fatal^**(^15^)**^.

In the present study, after a systematic review of the literature, we grouped the most common imaging findings of neurocryptococcosis, such as meningitis, hydrocephalus, and dilation of perivascular spaces, which are in line with the literature; and other less common findings, such as spinal cord lesions, C-IRIS, and cystic lesion with the dot sign. We also identified nonspecific patterns, which could be related to other etiologies concomitant with neurocryptococcosis, such as cortical atrophy, sinusitis, and infarcts, that do not necessarily have a direct relationship with the disease. Another important aspect is that there were 183 patients in whom the imaging examination did not demonstrate changes. Therefore, a normal examination does not rule out this diagnosis, given that more than one pattern can be seen in the same patient.

Our study has some limitations. Regarding the systematic review, it was evident that many authors do not describe the epidemiological characteristics of the populations studied; others simply exemplify the imaging findings without detailing information about the patients or categorizing them. Therefore, it was not possible to establish an accurate epidemiological profile of patients affected by neurocryptococcosis. Regarding imaging examinations, some articles used computed tomography scans alone, MRI scans alone, or both. It is known that some patterns are better described with certain methods, so it cannot be said that patients who were not subjected to both methods do not have the finding, because they may simply not have been diagnosed. Furthermore, some patients did not receive contrast, making the evaluation of the enhancement pattern of all findings limited. Some patterns were described by the authors in the works in a broad or subjective way, such as “T2 hyperintensities” or “focal hypointensities”, without representative images, making it impossible to fit them into specific categories.

## CONCLUSIONS

In view of the prevalence of neurocryptococcosis, the aim of this study was to understand the prevalence of different imaging patterns, mainly on MRI, exemplifying the most common patterns with specific images, in addition to rare patterns recently described in the literature and little known before, with the objective of improving the diagnostic accuracy and supporting clinical decision-making in uncertain scenarios.

After reviewing the literature, we can state that imaging examinations, especially MRI, are a valuable resource for the early diagnosis of cryptococcosis, assessment of its initial extent, and the pattern of the disease, especially in patients with neurological symptoms. However, the absence of neurological findings should not discourage the application of neuroimaging.
